# Design and Development of a Wearable Exoskeleton System for Stroke Rehabilitation

**DOI:** 10.3390/healthcare8010018

**Published:** 2020-01-15

**Authors:** Yang-Kun Ou, Yu-Lin Wang, Hua-Cheng Chang, Chun-Chih Chen

**Affiliations:** 1 Department of Creative Product Design, Southern Taiwan University of Science and Technology, Tainan 71005, Taiwan; ouyk@stust.edu.tw; 2 Department of Physical Medicine and Rehabilitation, Chi Mei Hospital, Tainan 71004, Taiwan; d8101080@gmail.com; 3 Center of General Education, Southern Taiwan University of Science and Technology, Tainan 71005, Taiwan; 4 Department of Biomedical Engineering, National Cheng Kung University, Tainan, Taiwan; 5 Department of Multimedia and Entertainment Science, Southern Taiwan University of Science and Technology, Tainan 71005, Taiwan; hcchang@stust.edu.tw; 6 Research and Development, AirTAC International Group, Tainan 74148, Taiwan

**Keywords:** stroke rehabilitation, wearable assistive device, exoskeleton, 3D printing

## Abstract

For more than a decade, many countries have been actively developing robotic assistive devices to assist in the rehabilitation of individuals with limb disability to regain function in the extremities. The exoskeleton assistive device in this study has been designed primarily for hemiplegic stroke patients to aid in the extension of fingers to open up the palm to simulate the effects of rehabilitation. This exoskeleton was designed as an anterior-support type to achieve palmar extension and acts as a robotic assistive device for rehabilitation in bilateral upper limb task training. Testing results show that this wearable exoskeleton assistive device with human factor consideration using percentile dimensions can provide comfortable wear on patients as well as adequate torque to pull individual fingers into flexion towards the palm for rehabilitation. We hope this exoskeleton device can help stroke patients with loss of function in the upper extremities to resume motor activities in order to maintain activities of daily living.

## 1. Introduction

There are two hundred million people all over the world suffering from loss of limb function [1], and most of these functions could be recovered with rehabilitation. Rehabilitation is mainly the use of other objects to force the affected limb to resume activity and has been shown in studies to aid in the paretic limb to recover [2]. Passive and consecutive activities can achieve the effects of physical therapy, can reduce muscle spasticity [3], and can stimulate activity in the cerebral cortex [4]. For more than a decade, many countries around the world are actively developing assistive devices using robotic technologies to help patients with loss of limb function due to various causes to undergo repetitive rehabilitation [5,6]: Jansen et al. designed a particular type of hybrid assistive limb exoskeleton for patients with spinal cord injury undergoing rehabilitation and underwent clinical trial with 21 patients; after training of 90 days, all patients showed significant improvement in their functional and ambulatory mobility without the exoskeleton [7]. Many researchers made lower extremity exoskeleton for gait rehabilitation [8,9,10,11] with various types of actuators such as regenerative magnetorheological actuator, series elastic actuator, electric motor actuator, etc. Some devices even enhance lower extremity performance [12,13,14,15] to provide better mobility to patients with knee injuries or other kinds of loss of function in the lower extremities. For the arms, many types of assistive exoskeleton device have been described [16,17,18,19,20,21], and most devices can be combined with other adapted equipment. However, only a few assistive exoskeleton rehabilitation devices for the hand have been described, mostly due to the complexity in the structure of the hand and the large range of motion that the fingers, making design for a hand assistive device very difficult. Bataller et al. [22] presented a design for a finger exoskeleton device with servomotors made from 3D printing that is low in cost and can be mass-produced for sports or rehabilitation for individual fingers. Iqbal et al. [23] described a hand exoskeleton rehabilitation device to facilitate tendon therapy exercises: this device covered only the proximal interphalangeal joint and utilizes the upward- and downward-movements of the said joint to bring about flexion and extension movements. Hence, the use of an exoskeleton assistive device for therapy of the individual with loss of limb function is a method that is both practical and convenient. Among the many causes of death, cerebrovascular disease places second in the world; colloquially known as “stroke,” it is the rupture of blood vessel in various parts of the brain and is one of the major causes of loss of limb function [24]. Stroke is defined by World Health Organization (WHO) as “rapidly developing clinical signs of focal (or global) disturbance of cerebral function, lasting more than 24 h or leading to death, with no apparent cause other than of vascular origin”. Common symptoms include weakness or numbness in one side of the face or of limbs, difficulty in swallowing or speech, vertigo, severe headache, hemiparesis, and loss of intellectual abilities. With recent advances in medicine, most stroke patients survive, but there is often damage to the motor neuron after the acute phase of the disease. It is found that 73–88% stroke survivors suffer the sequela of hemiparesis, accompanied by long-term loss of function [25,26]. Recovery after stroke depends on the different methods of rehabilitation as well as other treatments [27]. According to the American Heart Association (AHA), 55–75% of stroke patients suffer from upper limb dysfunction but persistent rehabilitation can usually recover partial function and only a few could attain complete recovery. The main reason only a few can recover is because most patients after stroke only rely on the unaffected side to perform normal daily activities. For example, before stroke occurred, an individual pours water from a pitcher with his right hand and drinks from the cup using the left. But after stroke occurred, his right arm became paretic so he switched to performing both the tasks of pouring and drinking with the left hand. As a result, what started as mere weakness in the right limb, after the transfer of all activities to the unaffected left limb, may eventually lose its function completely [28]. Past studies found that only 5–20% patients regain their upper limb functions; by one year after stroke, there are still 33% patients with no function in the upper limb—this shows the difficulty in upper limb rehabilitation. Normal upper limb function is a very important key in maintaining independent living; when the upper limb loses its function, activities of daily living are affected, thereby affecting the capability to live independently. This is also the reason behind the lack of patients’ participation in activities. Therefore, the recovery of upper limb function to restore normal activities is a very important issue [29,30].

In addition to exoskeleton assistive devices, recent years have also seen the development of methods specifically for limb rehabilitation, such as mirror therapy published in 1999 by Altschuler et al. to train upper limb function in stroke patients [31]: in this method, the paretic hand is kept inside a mirror-box while the mirror reflects the image of the normal, non-paretic hand, giving the illusion of it being the paretic hand. The visual effect from the mirrored reflection stimulates the premotor are of the brain as well as the posterior prefrontal cortex to engage the patient to perform activities in both hands simultaneously, which will in turn improve the rehabilitation of the paretic hand. This method has been demonstrated to be effective by many studies [32,33]. Furthermore, bilateral training of the upper limbs has also been shown to have a significant rehabilitative effect; studies have shown that, when compared with unilateral training, bilateral training can increase the frequency of training and that the effect is significantly better than unilateral training [34,35].

Therefore, this study hypothesizes the design of this particular exoskeleton rehabilitation device to achieve the following:

▪ for the healthy (non-paretic) hand to assist the paretic hand to undergo bilateral extension-flexion training simultaneously;
▪ for the exoskeleton rehabilitative device to allow also for the rehabilitation of the fingers;
▪ for the design of the exoskeleton assistive device to accommodate approximately 80–90% users;
▪ for the device to be light-weight, low-cost, and easy to fit onto the forearm.

## 2. The Human Hand Structure

**Figure 1:**
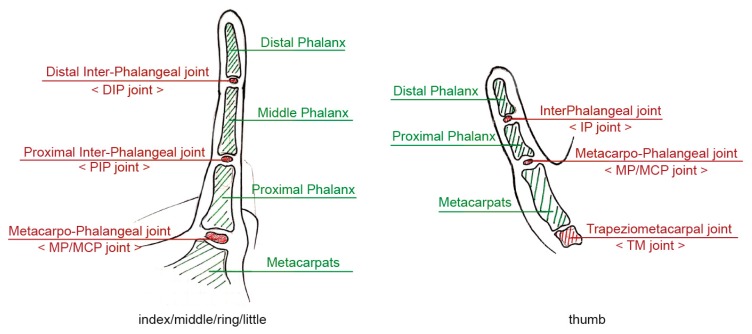
The human phalanx and finger joint.

The exoskeleton assistive device is to be worn directly over the hand; therefore, it must take into consideration the range of motion (ROM) and degrees of freedom (DOF) for each and every joint in the hand. With the exception of the thumb, every finger is made up of 3 joints and 4 bones—the joints of the fingers are metacarpophalangeal (MCP), proximal interphalangeal (PIP), and distal interphalangeal (DIP); the bones of the fingers are the metacarpals, proximal phalanx, middle phalanx, and distal phalanx. The thumb has no middle phalanx and is made up of two joints, metacarpophalangeal (MCP) and interphalangeal (IP). As shown in Figure 1, every MCP has two DOFs, while every PIP, DIP, and IP have one DOF, making up a total of 19 DOFs in each hand. The large number of DOFs makes any assistive device design for the hand quite challenging [36] and is made even more difficult by the complex structure of the bones of the hand: there is a great anatomical variation in the shape and dimensions of individual bones [37], the location on the device where the finger joint aligns is hard to accommodate to everyone’s hand size; for example, for the PIP, because of the variation in finger bone length, some may fall near the proximal phalanx while others fall near the distal, and the same scenario also applies to the DIP; while the MCP may not have this problem, because of the variation in palm width, the thumb is often either compressed or too far out and therefore often excluded from exoskeleton designs, making it hard to develop an exoskeleton for rehabilitation that can accommodate a large number of people.

## 3. Exoskeleton Structural Design

**Figure 2:**
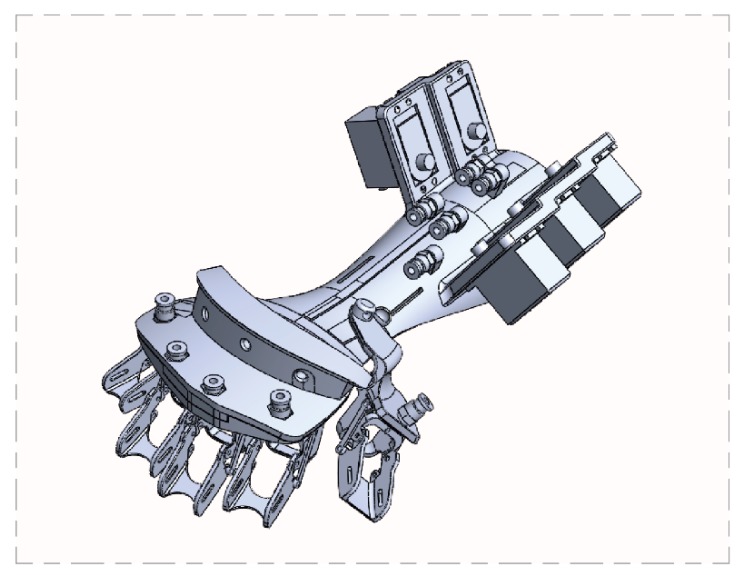
Schematic of the exoskeleton.

Current exoskeleton assistive devices on the market are fashioned as full skin coverage on the dorsal surface of the hand with a retractive design in which the palmar portion of the fingers are restrained with Velcro fasteners. When making a fist, the exoskeleton usually exerts force from the dorsal portion of the hand and, when extending the fingers, it uses external tension of the exoskeleton to pull on the Velcro fasteners to open up the palm, but this design is complicated by the aforementioned difficulty of varying lengths of finger segments, making it difficult to produce a single device that can fit all sizes. Furthermore, because of hypertonia (spasticity), the paretic hand of hemiplegic patients is clasped into a fist at resting state and it is easy for the hand to form a fist but extremely difficult to extend the fingers to open up the palm from a closed fist. It should be kept in mind that the main task in rehabilitation is to assist in allowing the fingers to perform extension and flexion at will. The exoskeleton assistive device presented in this study is designed mainly for hemiplegic stroke patients to simulate a rehabilitation therapy session to achieve finger extension. The exoskeleton is designed to exert force against the palmar surface of the hand to assist the patient in achieving finger extension. Figure 2 is a schematic diagram of the phalanx and finger joints of the exoskeleton. In this design, the phalanx part is made up of only the proximal and middle phalanx, and the PIP on the exoskeleton is where the proximal phalanx approximates the metacarpal bone. The exoskeleton PIP will align directly with the patient’s proximal phalanx; this ensures that, when the patient is wearing the device during therapy, the finger joint will align with the PIP. The exoskeleton DIP is where the middle phalanx approximates the distal phalanx and, for patients with shorter fingers, may end up aligning with the patient’s distal phalanx (rather than the DIP) but can nevertheless still achieve complete finger extension. Two sizes—M and L—are set to accommodate users with all glove sizes. The dimensions of our device were based on the Humanscale Manual [38], which contains over 60,000 bits of ergonomic and human engineering statistics for the human head, hands, and feet; is divided into ages 0.5–13 years and adults; and contains length, width, and angle dimensions from the 1st to the 99th percentiles. Dimensions for the M size of our device is based on the female 90th percentile data, whereas the L size is based on the male 90th percentile for individual angles, lengths, and finger joint widths of the five fingers of the hand. The exoskeleton thumb is designed as a detachable segment in order to accommodate different palm widths; as detailed in Figure 2, the detachable thumb is made with a movable joint that allows for thumb abduction and adduction and has various attachment sites to connect to the main body of the exoskeleton to adapt to different palm widths.

**Figure 3:**
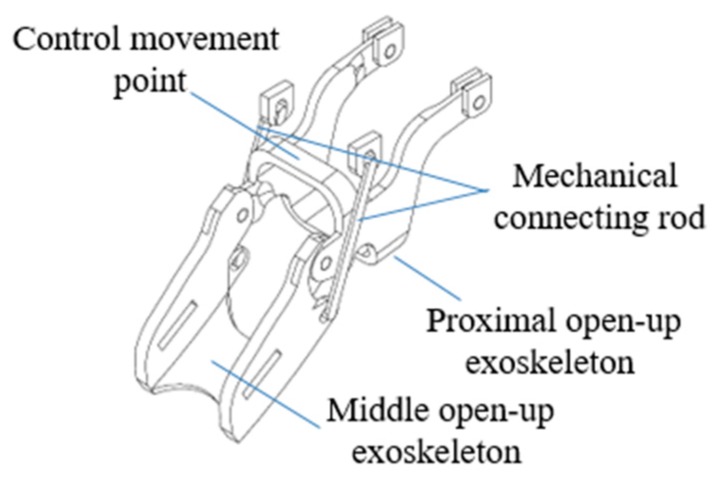
Schematic of the exoskeleton.

The exoskeleton interphalangeal joint is shown in Figure 3. The breadth of the five-finger joints was referenced using the largest male ring size. At MCP, the bending angle is set to be 0–70°, and at PIP, it is set at 0–90°. Because the mechanical pulling force is exerted only against the palmar surface, the patient’s finger flexion is unaffected, thus allowing for greater room for activity during therapy. Joints at 0° are equipped with safety baffle plates to ensure that the exoskeleton does not cause overextension of the fingers during therapy. The exoskeleton forearm was made with Poly Lactic Acid (PLA) material via 3D printing, with a total length of 290.10 mm and width of 121.87 mm, mainly to assist in finger movements of the hemiplegic arm. Every finger joint is equipped with a mechanical connecting rod, and there are 5 sets of servomotors to drive the connecting rod to control movement of every finger. The mechanical drive is on the middle phalanx where it approximates the PIP, but the main source of mechanical drive is still located at the PIP and the DIP is linked to the PIP via connected rods. Every finger uses one servomotor to achieve extension; when on the highest voltage of 7.4v, the drive is up to 37kg/cm. According to field testing, the process of movement is transmitted to the exoskeleton PIP and can provide a pulling force as high as 5 kg. Because, in the hemiplegic patient, the hand muscles have become rigid (spastic) and there may be varying degrees of hemiplegia as well as changes in the grip strength, the paretic hand is often clasped into a fist during therapy. Therefore a microcontroller module is necessary to control the servomotor with a larger torque, of which the internal control is programmed to 0° at the finger joint to serve as a limit control so that the motor will automatically stop when the angle of 0° has been achieved at the finger joint to avoid injury from overextension of the fingers. Also, there is an external emergency stop button for patients to press when they encounter any discomfort while using the device during a therapy session, which shuts off the power to the exoskeleton arm. The entire exoskeleton with the motors and electrical wiring weighs a total of 800 g.

### Static Analysis

**Figure 4:**
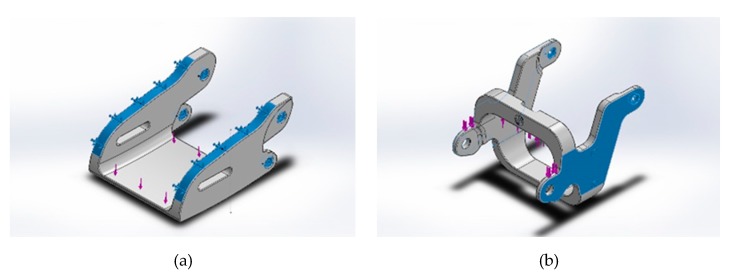
Fixed end in blue for (**a**) middle open-up exoskeleton and (**b**) control movement point.

Static analysis was performed using Solidworks on two components of the device: the middle open-up exoskeleton and the control movement point (please refer back to Figure 3) with the following configuration:

Material: Acrylonitrile Butadiene Styrene (ABS plastic)
Weight: middle open-up exoskeleton at 2.6 g; control movement point at 1.98 g
Boundary conditions: fixed end in blue (please see Figure 4)
Force exerted at blue: maximum downward force of 5 kgf, designated force of 2 kgf, and safety index 2.5
Grid: finite element analysis

**Figure 5:**
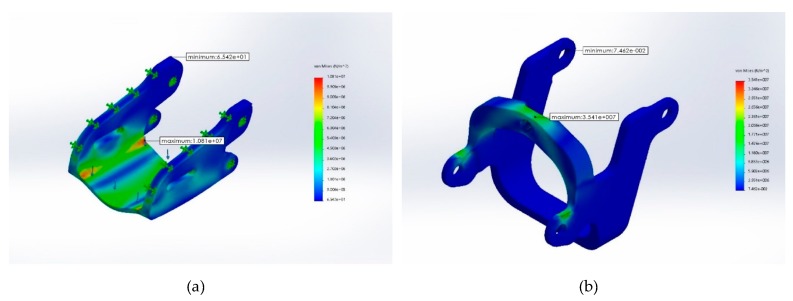
Stress distribution analysis for (**a**) middle open-up exoskeleton and (**b**) control movement point.

Results of stress distribution analysis (Figure 5) with stress concentrator at corner was 1.081 + 07 N/M^2^ approximating 1.103 kgf/mm^2^ for the middle open-up exoskeleton and 3.54123e + 007 N/m^2^ approximating 3.613 kgf/mm^2^ for the control movement point, inadequate to cause structural collapse for either component.

**Figure 6:**
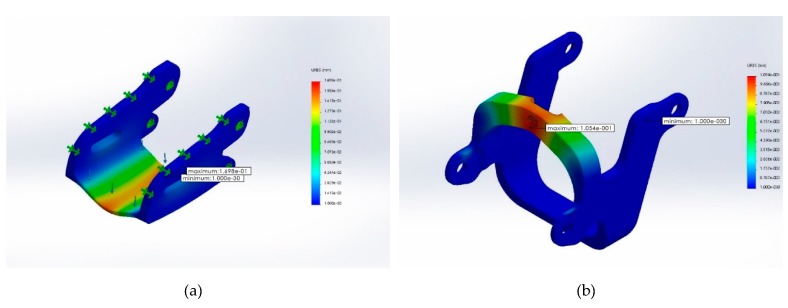
Strain analysis for (**a**) middle open-up exoskeleton and (**b**) control movement point.

Strain analysis (Figure 6) showed maximum deformation to be 0.16 mm for the middle open-up exoskeleton and 0.11 mm for the control movement point, inadequate to cause structural collapse for either component.

## 4. Electromechanical Integration Design

**Figure 7:**
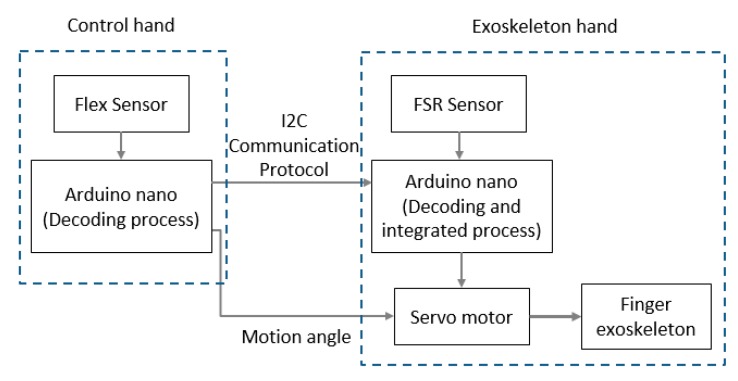
Schematic of signal transmission.

The exoskeleton assistive device in this study also includes a neoprene rehabilitation assistive glove to be worn on the non-paretic hand. Each fingertip on the glove is fitted with a set of strain gauge module (BF350-3AA), which functions mainly to extract the bending angle data during finger extension-flexion of the non-paretic hand and to relay the signal back to the microcontroller module (TI-MSP430). Through an algorithm, the bending angle of each finger in the non-paretic hand is sent via Wi-Fi to the exoskeleton to set the servomotor in motion to transmit the corresponding degree of electrical power to pull on the connecting rods on the device in order to bring about movements in the paretic hand to mimic those of the non-paretic hand while simultaneously collecting signals from the sensors to allow the mimicking movements to occur simultaneously with the non-paretic hand, achieving the effect of mirror therapy in the upper limbs. Figure 7 illustrates the signal transmission. Movements of the upper limb is reconstructed using an algorithm through signal filtering sequence to exclude noise from background and unintentional movements. Feature extraction is used to draw out the feature of each movement in mirror therapy, and feature reduction is used to scale down the computational complexity and to augment movement discrimination. For signal filtering, in order to lower the high-frequency noise error of the signal of the acceleration and the angular velocity, the calibrated signal needs to go through a low-pass filter (for example, moving average filter, Butterworth filter, or Chebyshev filter) to filter high-frequency noises.

### 4.1. Acceleration and Velocity

**Figure 8:**
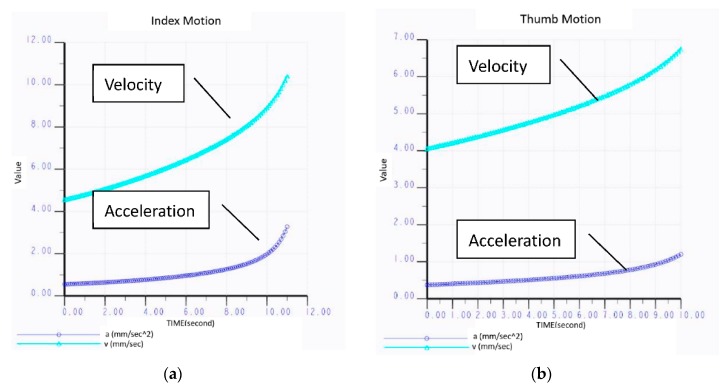
(**a**) Acceleration of exoskeleton index finger. (**b**) Acceleration of exoskeleton thumb.

If the motor angular velocity remains constant (for example, 2 deg/s) and consistent with the speed transmitted to the part of the device in contact with a user’s hand, it would take 10 s for the proximal end of the index finger to achieve full extension from a flexed position, before the velocity increases steadily (Figure 8a), while the thumb would take 9.5 s (Figure 8b), before the velocity also increases steadily.

### 4.2. Force Sensitive Resistor Sensor

**Figure 9:**
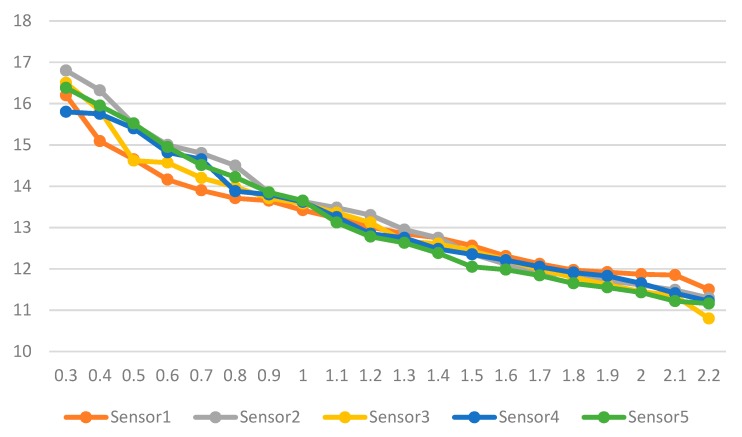
Force sensitivity analysis.

Five sensors were installed to measure force sensitivity of the hands against the exoskeleton device and found that resistance and force have an inverse and linear relationship with the R^2^ for the five sensors ranging between 0.9213 and 0.9588, as shown in Figure 9, where the y-axis is the force sensing resistance in Ohms and the x-axis is the force in kgf.

## 5. Operating Analysis

### 5.1. Parameter Definitions

**Figure 10:**
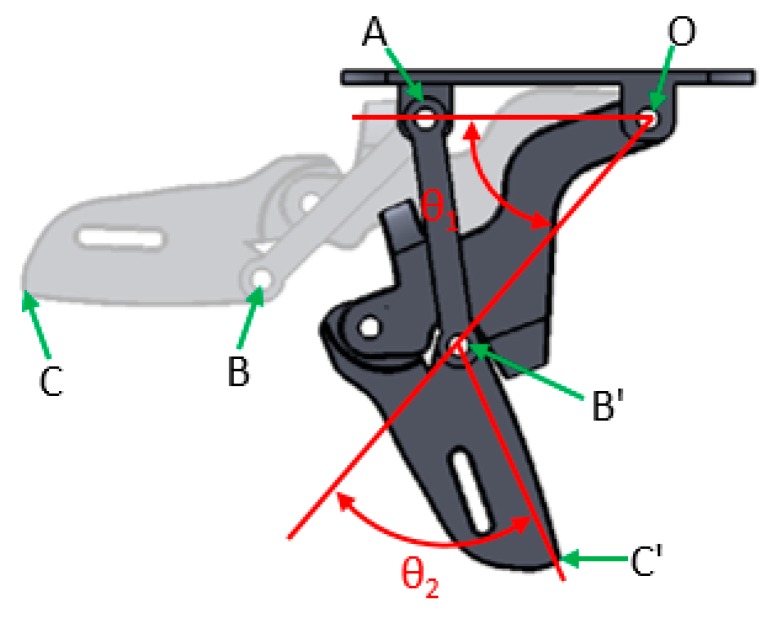
Schematic of finger joint parameters.

The exoskeleton in this study includes only the proximal and middle phalanges; the parameters of the finger joint are defined as shown in Figure 10. Point O is the point where the lower-support joint attaches to the core of the exoskeleton, akin to the MCP joint of the hand, and therefore serves as the origin of the coordinates where the X and Y axes are both zero. Point A is the fixing point for the connecting rod where the relationship between the MCP and PCP is controlled; point B is the position of the PIP at rest, while B’ is the shifted PIP position after movement. Similarly, C is the position of DIP at rest, while C’ is the shifted DIP position after movement. Point C is the terminal end of the exoskeleton when it moves the connecting rod moves with it and affects the rest of the finger joints. $\bar{\mathrm{OB}}$ is the proximal phalanx, $\bar{\mathrm{BC}}$ is the middle phalanx, $\theta_{1}$ is the DOF of the MCP as well as change in MCP angle, whereas $\theta_{2}$ is that of PIP. The relationships between every point, line, and angle are known, and every phalangeal joint has only one DOF, with a total of 10 DOFs. Therefore the actuation of the device is movement on a level plane, where finger joint movement is brought about using mechanical connecting rods; therefore, $\theta_{2}$ changes in accordance with $\theta_{1}$ and the angles and positions of each finger movement of the patient can be indicated using the actuation tracks of points O, B, and C.

### 5.2. Analysis of Movement

The relationship between points O and B is a linear one and between points B and C; $\bar{\mathrm{OB}}$ and $\bar{\mathrm{BC}}$ are indicated with the distance formulae below:

(1) $$\bar{\mathrm{OB}}=\sqrt{{\left(X_{B}-X_{O}\right)}^{2}+{\left(Y_{B}-Y_{O}\right)}^{2}}$$

(2) $$\bar{\mathrm{BC}}=\;\sqrt{{\left(X_{C}-X_{B}\right)}^{2}+{\left(Y_{C}-Y_{B}\right)}^{2}}$$

Whereas the position of $B^{\prime }$ dictates movement changes of $\bar{\mathrm{OB}}$, $B^{\prime }$ is B multiplied by the rotation matrix of $\theta_{1}$, of which the formula is denoted by Rot(θ) as follows:

(3) $$B^{\prime }=B\left(x,y\right)\;\times \;\mathrm{Rot}\left(\theta_{1}\right)$$

The movement pattern by $C^{\prime }$ is more complex: as shown in Figure 5, movement of C simultaneously affects the parameters of $\theta_{1}$, ${\;\theta }_{2}$, and point B, and its position is in turn altered when B transforms into $B^{\prime }$. The relationship between points $C^{\prime }$ and O is formulated below:

(4) C′O(x, y)=B′O(x,y)+RotO(θ1)×C′B′(x,y)

Since $C^{\prime }$ is the terminal end of the exoskeleton assistive device, its movement affects other parameters the most and its post-movement position can be used to back-trace positions and angles of other points.

## 6. Test Results

### Simulated Results

The relationship formulae between the points can be used to obtain the dimensions of the various parts of the exoskeleton assistive device; and with the aforementioned range of bending angle in MCP and PIP, the position and angle of individual points during movement could be simulated and compared to actual measurements.

**Figure 11:**
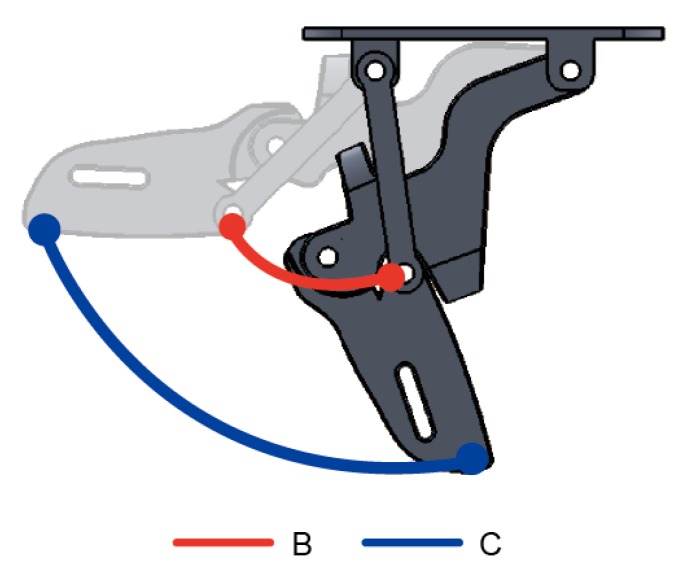
Schematic of simulated finger joint movement arc.

Figure 11 is the scatterplot based on alternating between the minimum and maximum angles of $\theta_{1}$ from 0~70°. Post-movement points $B^{\prime }$ and $C^{\prime }$ computed from Equations (3) and (4) using the positions of B and C and the varying angles of $\theta_{1}$ are compared to those from the outer appearance based on Solidworks design layout. The positions of $B^{\prime }$ and $C^{\prime }$ simulated from the formulae as a result of movement matched completely with the movement arc on the design layout.

**Figure 12:**
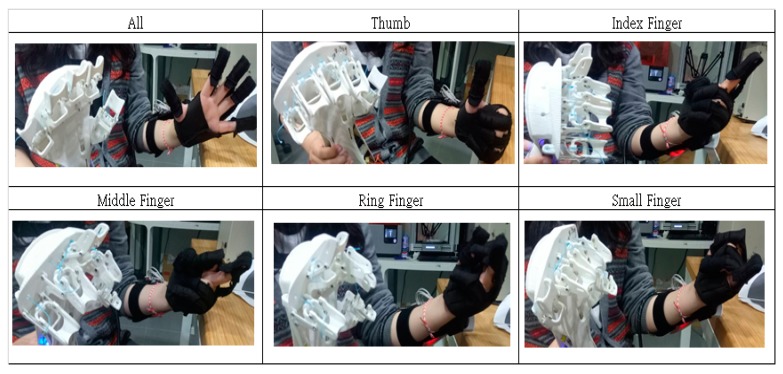
Actual movements of each finger joint.

Figure 12 demonstrates the actual movement of individual fingers. In this demonstration, the assistive glove is worn on the left hand while the exoskeleton device is on the right. The bending angle of fingers in the left hand drives varying degrees of bending, and the fingers can move in a precise manner during simulation. However, while individual fingers are moving, other fingers also bend slightly—this phenomenon is most apparent in the ring finger and is caused by the connectedness among tendons in the human hand and is part of a normal finger reaction. It also shows that the bending angle algorithm can gauge in a precise manner the changes in the hand when bending and can respond appropriately.

**Figure 13:**
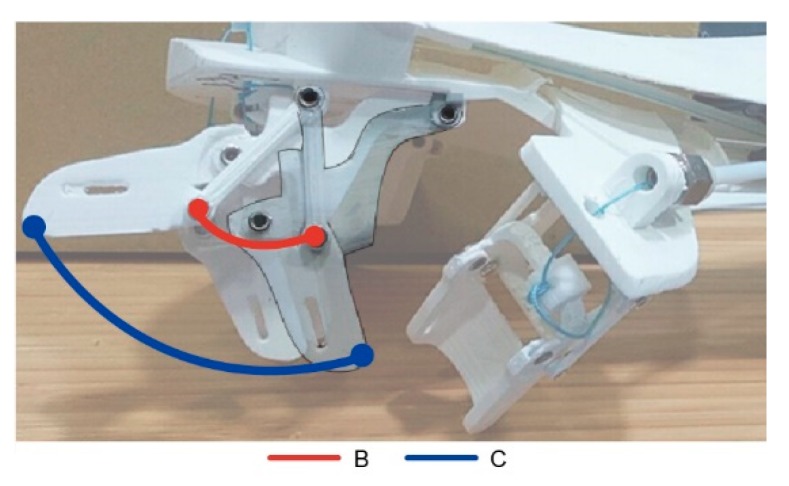
Actual movement of the finger joint arc.

When comparing the tracks from movements of the hand with formulae-derived position points of each phalangeal joint, as an example illustrated in Figure 13, it can be seen that the movement arcs for points $B^{\prime }$ and $C^{\prime }$ are identical to those from operating the assistive glove. Since the use of different methods to validate the movement patterns result in the same movement arc, it therefore confirms the appropriateness and the practicality of the constructs in this study. What should also not be overlooked is that it provides greater DOFs to the finger joints and its cost is cheaper compared to other exoskeleton devices on the market. The range of motion in other existing exoskeleton models fall between 0~55° for MCP and 0~65° for PIP [39]; in contrast, the exoskeleton in this study offers 0~70° for MCP and 0~90° for PIP and is contrary to other designs that exclude the thumb or utilize a fixed thumb [39,40]. The exoskeleton in this study offers a range of motion up to 35° for the thumb—a greater angle means more room for motion and can provide better rehabilitation results for stroke patients with upper limb hemiplegia, as detailed in Table 1.

## 7. Discussion and Conclusions

The design concept of the exoskeleton assistive device in this study stems from multiple medical studies for the rehabilitation of hemiplegic stroke patients; therefore, it can provide a better therapeutic effect in the rehabilitation process. The assistive device weighs only around 800 g in its entirety; is portable; provides a more powerful torque to pull on the fingers; and can accommodate hemiplegic stroke patients with varying degrees of disease severity, differing palm sizes, differing finger segment lengths, and differing finger breadth to cater to most patients. As a whole, this device is more than capable of achieving therapeutic goals in addition to being safe and convenient to use and can easily be adapted for general use. 

Currently, the proofing of all parts for testing purposes brings the cost to within $650 USD, which is lower than the market price; furthermore, other current exoskeleton rehabilitation devices are mostly used in rehabilitation institutions, while the device in this study offers hemiplegic stroke patients the option to undergo rehabilitation in the comfort of his or her own home and anticipates to improve further on the portability, safety, and cost to allow patients to use the device at home for self-rehabilitation.

This paper describes the design of an exoskeleton assistive device for the hand based on principles of mirror therapy with an innovative design, in which finger movements are powered from the palmar side (hence, the term “lower-support type”) and was made from 3D printing while able to retain structural integrity as demonstrated by static analysis and force sensitivity analysis. Three-dimensional printing is low in cost and therefore could easily be made widely accessible; therefore, this device can offer the most benefit at a reduced cost for upper extremity rehabilitation and hereafter can improve the function and the quality of life of patients.

Many current studies of exoskeleton rehabilitation devices remain at the testing level in institutions and cannot capture problems and difficulties encountered in real-life use, but the assistive device in this study has already worked with several hospitals for on-site testing and is in the process of improving the structural design using feedback from real-life testing.

Stroke rehabilitation is a rather dull process that is ongoing and repetitive, making it difficult for patients to go through the entire process with patience. In order to motivate stroke patients to actively participate in the rehabilitation process, further developments may see the addition of VR (virtual reality) elements to enrich the rehabilitation experience to speed up patient recovery. Because VR can incorporate entertaining game themes, can increase the level of attention in stroke patients during therapy, can reduce the sense of loss from the loss of function due to the disease, and is significantly more effective compared to conventional therapy [41,42], it is set to be the next direction of this study.

## Figures and Tables

**Table 1:** Comparison of exoskeleton rehabilitation devices.

Labels	MCP	PIP	DIP	Transverse DOF—Thumb	Weight	Clinical Testing
The design in this study	70°	90°	N.A.	35°	800g	No
Susanto et al., 2015 [39]	55°	65°	N.A.	N.A.	>1kg	Yes
Pu et al., 2014 [40]	90°	80°	100°	N.A.	700g	No

Note: MCP = metacarpophalangeal joint; PIP = proximal interphalangeal joint; DIP = distal interphalangeal joint; DOF = degree of freedom.

## Abbreviations

DIP	distal interphalangeal joint
DOF	degree of freedom
IP	interphalangeal joint
MCP	metacarpophalangeal joint
PIP	proximal interphalangeal joint
ROM	range of motion
